# Intergenerational breastfeeding practices among parents and children: 1993 Pelotas (Brazil) birth cohort

**DOI:** 10.1111/mcn.13058

**Published:** 2020-07-06

**Authors:** Juliana dos Santos Vaz, Leonardo Pozza dos Santos, Giovanna Gatica‐Dominguez, Isabel Oliveira Bierhals, Ana Paula Gomes, Helen Gonçalves, Gilberto Kac, Ana Baptista Menezes, Maria Cecilia Formoso Assunção

**Affiliations:** ^1^ Postgraduate Program in Epidemiology Federal University of Pelotas Pelotas Brazil; ^2^ Faculty of Nutrition Federal University of Pelotas Pelotas Brazil; ^3^ Nutrition College Federal University of Pampa Itaqui Brazil; ^4^ Municipal Secretary of Health Pelotas Brazil; ^5^ Institute of Nutrition Federal University of Rio de Janeiro Brazil; ^6^ Rio Grande University Foundation Rio Grande Brazil

**Keywords:** birth studies, breast feeding, child, infant, parents, prospective studies

## Abstract

The objective of this study was to investigate intergenerational breastfeeding practices according to parental sex and age at delivery in the 1993 Pelotas (Brazil) birth cohort study. This is a prospective birth cohort study, and at the 22‐year follow‐up, a substudy with all children of the cohort members who had become parents was conducted (93Cohort‐II). First generation breastfeeding data were collected at 3 months and 4‐year‐old follow‐ups. In the 93Cohort‐II, parents answered a questionnaire about their children's breastfeeding practices. Adjusted Tobit and Poisson regression models with robust variance were applied to estimate the association between predominant parental breastfeeding duration and exclusive breastfeeding duration of the children at 3 and 6 months. Out of 3,810 cohort participants, 955 (25%) had delivered at least one live‐born infant, and 1,222 children were assessed. Fifty‐four percent of parents were ≤19 years old. Direct effects of predominant parental breastfeeding duration on exclusive breastfeeding duration of their children were only observed when data were stratified by parental age: children born to parents aged ≥20 years old and who were predominantly breastfed for at least 3 months presented higher exclusive breastfeeding duration and higher prevalence of being exclusively breastfed for at least 3 months. When analyses were stratified by mothers and fathers, the result remained significant only among mothers. Longer predominant breastfeeding duration in the first generation was associated with longer exclusive breastfeeding duration in the second generation, but only among older mothers. Education and social support surrounding breastfeeding should be intensified among fathers and younger parents to create a positive environment supportive of breastfeeding.

## 1 INTRODUCTION

The benefits of breastfeeding for child health and development are widely known. In the short term, breastfeeding protects against infectious diseases in childhood (Victora et al., 1987), whereas in the medium and long term, it improves intelligence, reduces the risk of non‐communicable diseases and affects both the individual and social level by increasing educational attainment and earnings (Victora et al., 2015). Despite widespread knowledge about breastfeeding importance, increasing the duration of breastfeeding and achieving exclusive breastfeeding for at least 6 months still is a challenge worldwide (Victora et al., 2016).

There are many factors that enable breastfeeding practices at the structural level (i.e., the breast milk substitute market and marketing strategies, and the sociocultural context) and the environmental level (health systems and services, family and community and workplace and employment). At the individual level, women's breastfeeding behaviour is influenced by personal characteristics such as age, nutritional status, education and confidence, as well as by their babies' characteristics such as sex, wellbeing and temperament. Breastfeeding is a behaviour that entails a mother–baby relationship (Rollins et al., 2016).

Cultural differences between geographical regions play an important role in the determination of breastfeeding practices in Brazil. However, few national studies have explored this matter. A study with data from the 2002–2003 Brazilian Family Budget Survey identified the factors associated with cessation of breastfeeding before 6 months of age. Those factors were having four or more residents living in the same household and mothers aged 30 years or more. Conversely, factors that positively influenced higher breastfeeding duration were higher socio‐economic level, families with two or more children under 5 years old and living in rural area (Wenzel, Ocaña‐Riola, Maroto‐Navarro, & de Souza, 2010).

Mean maternal age at first birth in 2010 was 24 years old and had barely changed in Brazil since 1980 (Miranda‐Ribeiro, Garcia, & Faria, 2019). In a prospective study using data from five birth cohorts from low‐ and middle‐income countries (LMICs), a positive association between maternal age and breastfeeding duration was found. The authors concluded that children of young mothers (≤19 years old) in LMICs are disadvantaged at birth and in childhood regarding nutrition and schooling when compared with mothers aged 20–24 years (Fall et al., 2015).

Evidence suggests a possible intergenerational repetition of breastfeeding practices, but the mechanisms could not be fully elucidated. Thus, the authors recommended performing more consolidated prospective cohort studies to examine the mechanisms that promote the intergenerational practice of breastfeeding (Di Manno, Macdonald, & Knight, 2015). Intergenerational repetition of breastfeeding duration was investigated among the offspring of 420 adolescent women (19 years old or younger) belonging to the 1982 Pelotas birth cohort study. The authors observed a prevalence ratio (PR) of 29% of stopping breastfeeding at 6 months of age in mothers who were breastfed for less than 1 month (Horta, Victora, Gigante, Santos, & Barros, 2007).

Nevertheless, the 1982 cohort study included only adolescent mothers in their analysis and did not explore this relationship in adult women whose sense of responsibility to look after a child may differ compared with young mothers. The intergenerational breastfeeding practice among fathers as part of their offspring's well‐being was also unexplored. To adequately address public policies and programmes to protect, support and promote breastfeeding, it is important to understand the factors associated with the duration of breastfeeding, either exclusive or partial. Therefore, the objective of this study was to investigate intergenerational breastfeeding practices according to parental sex and age at delivery in the 1993 Pelotas birth cohort study. Older mothers who were predominantly breastfed for at least 3 months were 40% more likely to exclusively breastfed their children for at least 3 months.

## 2 METHODS

### 2.1 The 1993 Pelotas birth cohort

The 1993 Pelotas birth cohort study is a population‐based birth cohort that was launched in 1993 in the city of Pelotas, southern Brazil. All mothers delivering live births (*n* = 5,265) from January 1 to December 31, 1993 in Pelotas' five maternity hospitals were invited to participate in the study. Sixteen mothers refused to participate, and a total of 5,249 mothers of eligible newborns were interviewed in the first 24 h after delivery.

**FIGURE 1:**
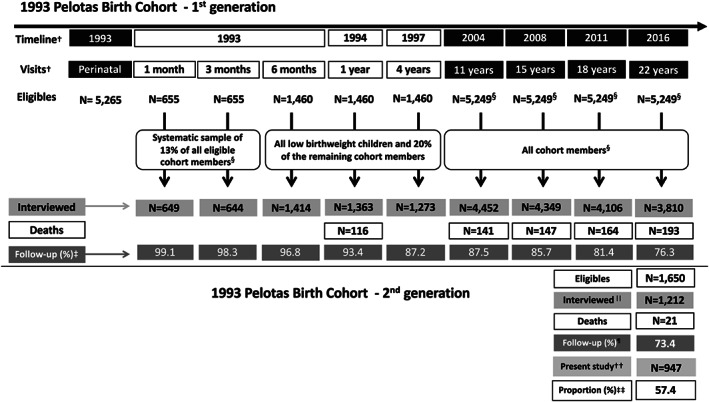
Flow chart of the first and second generation of the 1993 Pelotas birth cohort study. ^†^Black boxes indicate follow‐ups that all cohort members were eligible to participate. ^§^Eligibility refers to 5,249 out of 5,265 cohort members whose mothers provided consent to participate in the recruitment phase (perinatal). Mothers of the remaining 16 refused to participate. ^‡^First generation proportion of completed follow‐up considering members who participate, added to those known to have die; ^||^1,212 refer to the second generation children with completed assessments at the research clinic unit. A further 44 children received home visits, and parents answered a brief questionnaire. ^¶^Second generation proportion of completed follow‐up considering children who participate. ^††^Final sample of second generation children, starting from a number of 1,222 children with available breastfeeding data, excluding those who were still being exclusively breastfed (*n* = 203) and those whose exclusive breastfeeding duration was greater than 6 months (*n* = 72). ^‡‡^Present study proportion of participants, considering 947 children in relation to the total eligible

Several follow‐ups of this cohort have been performed since then. Follow‐ups that happened before cohort members were 11 years old only examined cohort subsamples (all low birth weight children plus 20% of the remaining cohort, who were randomly selected). In the follow‐ups at 11, 15, 18 and 22 years of age, the whole cohort was invited to participate. In all these visits, the proportions of completed follow‐up were greater than 75%, and trained interviewers collected information on socio‐economic and demographic characteristics, cognitive development and anthropometry and nutritional status (Figure 1). Complete information about the perinatal study and all follow‐up waves has been published previously (Gonçalves et al., 2014; Gonçalves et al., 2018; Victora et al., 2008).

### 2.2 The 22‐year‐old follow‐up and second generation recruitment

In 2015, when participants were 22 years old, they received an invitation to attend follow‐up assessment at the research clinic. Participation in this follow‐up involved 3,810 individuals (76% of the original sample), and data on anthropometry, body composition and body shape, lifestyle, feeding habits, physical activity and health status were collected.

Out of the 3,810 individuals, 955 (25%) reported having had at least one child. These 955 cohort members who had become parents were then invited to take part in a substudy with their children, the so‐called second generation of the 1993 Pelotas birth cohort study (93Cohort‐II). Of the 1,650 children identified, 21 died before the cohort follow‐up, and 373 were considered losses. Of the remaining 1,256 children, 1,212 were studied along with their parents at the research clinic unit. A further 44 children were unable to attend the clinic, so trained interviewers conducted home visits, and a short version of the original questionnaire was applied to their mothers and/or fathers. In this study, 1,222 children presented complete breastfeeding data. We excluded 203 children who were still being exclusively breastfed at the time of the interview and 72 children whose exclusive breastfeeding duration was greater than 6 months, because this information is potentially inconsistent. The final sample consisted of 947 (57%) children of 93Cohort‐II and 759 parents (80%) (Figure 1).

### 2.3 Breastfeeding practices of parents and children

Data about breastfeeding duration in the first generation of the 1993 Pelotas birth cohort study were collected when individuals were between 3 months and 4 years old. As exclusive breastfeeding was barely practiced in the first generation, we used data on predominant breastfeeding duration (main exposure). This procedure has been used as an alternative to explore breastfeeding practices in the 1982 and the 1993 Pelotas birth cohorts (Neutzling et al., 2009; Orlandi et al., 2017; Victora et al., 2015). We defined predominant breastfeeding duration as the total time that children's feeding was based on breast milk, infusions or water with no addition of semi‐solid or solid foods.

In the 93Cohort‐II, parents were asked if their children were breastfed and how old their children were when they stopped receiving breast milk alone, that is, without additional water or infusions. Based on this question, we defined exclusive breastfeeding duration (main outcome) as the total time that children's feeding was solely based on breast milk, with no additional infusions, water or any other liquid. In the 93Cohort‐II, parents were also asked for how long (in months and days) their children had been exclusively breastfed, and two categorical variables were generated, which allowed us to assess the prevalence of exclusive breastfeeding at 3 and 6 months of age. The prevalence of exclusive breastfeeding for 3 or 6 months was calculated by dividing children exclusively breastfed for, at least, 3 or 6 months by the total number of children included in analyses and multiplied by 100.

### 2.4 Multiple imputation process

As described above, only subsamples of the cohort's first generation were followed until cohort members reached 11 years of age. As a result, information about predominant breastfeeding was available for only 293 out of 955 parents. To overcome this issue, we performed a multiple imputation process for those 662 parents who had no available information about predominant breastfeeding duration.

Multiple imputation using the chained equation approach was used to impute all missing parental breastfeeding information, using the command *mi impute chained (reg)* in Stata. The imputation model included the variable to be imputed (predominant parental breastfeeding duration) and exclusive breastfeeding duration of children, family income in 1993, maternal education and skin colour, parent's sex and age, type of delivery, preterm birth status and birth weight of the second generation (potential confounders of the model, as described below). As the missing information on the predominant breastfeeding duration of the first generation was not at random, that is, low birth weight cohort members were overrepresented, we also included birth weight of the first generation in the multiple imputation model.

Stability of the imputation process was determined taking into account the average difference between the regression coefficients calculated from the complete and the imputed data (the bias), as well as the standard error of the bias. Five data sets were generated as we noted that after five cycles, the imputation process was found to be stable, that is, the bias and the standard error of the bias have remained close to zero. Results for predominant breastfeeding duration were calculated by averaging all five imputed data sets. All the information about the imputation process can be assessed in Tables S1 to S5.

### 2.5 Statistical analysis

We described second generation exclusive breastfeeding duration using mean and standard deviation and median and interquartile range. Differences between second generation exclusive breastfeeding duration according to parent's and children's characteristics were assessed using analysis of variance.

The association of first generation predominant breastfeeding duration with second generation exclusive breastfeeding duration was assessed using Tobit regression, as we censored exclusive breastfeeding distribution at 6 months. Confounders of this association were defined using a directed acyclic graph (DAG) (Figure S1). We developed the DAG from a primary set of variables to identify a minimum and sufficient number of potential confounders using the DAGitty software (Textor, 2015). The following variables were included in the DAG: (i) cohort members' characteristics (first generation): sex, skin colour, birth weight, preterm birth (<37 weeks), type of delivery (normal or caesarean section), family income at birth, maternal age and education at delivery and maternal pre‐pregnancy body mass index (BMI); (ii) second generation characteristics: birth weight, preterm birth (<37 weeks), type of delivery (normal or caesarean section), maternal age at delivery and hospitalization.

According to the DAG, the minimal sufficient adjustment set to estimate the association between first generation predominant breastfeeding duration and second generation exclusive breastfeeding duration comprised the following variables: first generation family income at birth, maternal education at delivery, skin colour, age and sex; second generation type of delivery, preterm birth status and birth weight. We also included first generation birth weight in the tested models because the missing information on breastfeeding was not at random and this variable was important in the imputation process.

Finally, to assess the magnitude of predominant breastfeeding duration association with the prevalence of having been exclusively breastfed for at least 3 and 6 months in the second generation, we used Poisson regression with robust variance, as the PR is easier to interpret than the odds ratio (Barros & Hirakata, 2003). Here, we also adjusted the analysis using the same set of confounders included in the Tobit regression models.

To test for possible differences in breastfeeding practices between adolescent and adult parents, we performed Tobit and Poisson regression analyses stratifying according to parental age (≤19 and ≥20 years). Additionally, all analyses were also stratified by parental sex.

All analyses were conducted using Stata Data Analysis and Statistical Software (STATA) version 15.0 (Stata Corp., College Station, Texas, USA). Collinearity was assessed in all tested models using the variance inflation factor. Results stratified using imputed and nonimputed data were also tested to detect possible bias in the imputation process. Results are presented in the Supporting Information.

### 2.6 Ethical considerations

This study was conducted according to the guidelines laid down in the Declaration of Helsinki, and all procedures involving research study participants were approved by the Research Ethics Committee of the Federal University of Pelotas School of Medicine (Protocol number: 1.250.366). Written informed consent was obtained from all participants before each follow‐up.

## 3 RESULTS

The 759 parents included in the analyses were more likely to be women (75%), and 39% of them were born in the lowest tertile of the socio‐economic strata. Twelve percent of them had low birth weight, and 54% were under 19‐year‐old when their child was born. Children were more likely to be boys (53%), almost 85% of them were their parents' first child, 18% were 12 months old or younger, 10% had low birth weight and 10% were premature. More than half of the second generation children were born by C‐section (Table 1).

**TABLE 1:** Parents' and children's characteristics according to breastfeeding of the first child

Variable	*N* (%)
Parents' characteristics (*n* = 759)	
Parentage	
Mother	570 (75.1)
Father	189 (24.9)
Family income in 1993 (tertiles)	
First (lowest)	291 (39.4)
Second	195 (26.4)
Third (highest)	252 (34.2)
Age at delivery (years)	
≤19	408 (54.0)
≥20	348 (46.0)
Low birth weight (<2,500 g)	
No	615 (88.0)
Yes	84 (12.0)
Children's characteristics (*n* = 947)	
Sex	
Male	505 (53.3)
Female	442 (46.7)
Birth order	
First	800 (84.5)
Second or after	147 (15.5)
Age (months)	
≤12	173 (18.3)
>12	774 (81.7)
Low birthweight (<2,500 g)	
No	830 (90.4)
Yes	88 (9.6)
Preterm birth (<37 gestational weeks)	
No	841 (89.3)
Yes	101 (10.7)
Type of delivery	
Normal	457 (48.5)
C‐section	485 (51.5)

*Note.* Second generation of the 1993 Pelotas (Brazil) birth cohort study.

Mean predominant breastfeeding duration among members of the 1993 Pelotas cohort study who had at least one child by the 22‐year‐old follow‐up was 1.7 months, with no differences according to sex. The mean exclusive breastfeeding duration was 3.4 months for the second generation. In addition, while 10% of mothers and fathers were predominantly breastfed for at least 3 months, more than 60% of second generation children were exclusively breastfed for at least 3 months (data not shown).

Mean second generation exclusive breastfeeding duration did not differ according to parental sex, family income in 1993, parent's birth weight, child's sex, birth order, preterm birth and type of delivery. Nevertheless, the duration of exclusive breastfeeding was lower among children of older parents, who were ≤12 months old and had low birth weight (Table 2).

**TABLE 2:** Exclusive breastfeeding duration of the second generation of the 1993 Pelotas (Brazil) birth cohort study

Variable	Exclusive breastfeeding duration in months	
	Mean (95% CI)	Median (IQR)
Parents' characteristics (*n* = 947)		
Parentage	*0.420*	
Mother	3.5 (3.3; 3.6)	4 (2–6)
Father	3.3 (3.0; 3.6)	3 (1–6)
Family income in 1993 (tertiles)	*0.138*	
First (lowest)	3.6 (3.3; 3.8)	4 (2–6)
Second	3.4 (3.2; 3.7)	3 (2–6)
Third (highest)	3.2 (3.0; 3.5)	3 (1–6)
Age at delivery (years)	*0.034*	
≤19	3.6 (3.4; 3.8)	4 (2–6)
≥20	3.3 (3.1; 3.5)	3 (1–5)
Low birth weight (<2,500 g)	*0.099*	
No	3.5 (3.3; 3.6)	4 (2–6)
Yes	3.1 (2.7; 3.5)	3 (1–5)
Children's characteristics (*n* = 947)		
Sex	*0.763*	
Male	3.4 (3.2; 3.6)	4 (1–6)
Female	3.5 (3.3; 3.6)	3 (2–6)
Birth order	*0.137*	
First	3.5 (3.3; 3.7)	4 (2–6)
Second or after	3.3 (3.0; 3.5)	3 (2–5)
Age (months)	*<0.001*	
≤12	2.6 (2.3; 3.0)	2 (1–5)
>12	3.7 (3.5; 3.8)	4 (2–6)
Low birth weight (<2,500 g)	*0.025*	
No	3.5 (3.3; 3.6)	4 (2–6)
Yes	2.9 (2.4; 3.4)	3 (1–6)
Preterm birth (<37 gestational weeks)	*0.424*	
No	3.4 (3.3; 3.6)	4 (2–6)
Yes	3.3 (2.8; 3.7)	3 (1–6)
Type of delivery	*0.762*	
Normal	3.5 (3.3; 3.6)	3 (2–6)
C‐section	3.4 (3.2; 3.6)	4 (1–6)

*Note. P* values refer to analysis of variance (ANOVA).

Abbreviations: 95% CI, 95% confidence interval; IQR, interquartile range.

**FIGURE 2:**
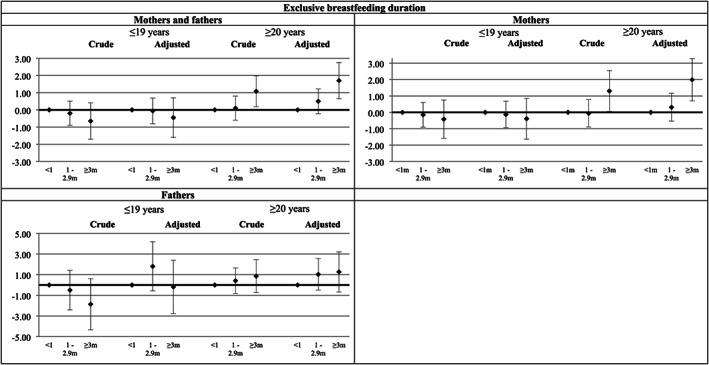
Association between first generation's predominant breastfeeding duration and second generation's exclusive breastfeeding duration measured by Tobit regression. Note: adjusted for family income in 1993, grandmother's education and skin colour, maternal/paternal birthweight, age and sex, and second generation delivery type, prematurity, and birthweight

The Tobit regression showed no association between parental predominant breastfeeding duration and exclusive breastfeeding duration of their children (Table 3). Nonetheless, when analyses were stratified for parental age at birth, a direct association of parental breastfeeding duration with child breastfeeding was observed for older parents, that is, those parents who were 20 years old or older by the time of delivery and had been predominantly breastfed for at least 3 months, exclusively breastfed their children for a longer period when compared with those parents who were predominantly breastfed for less than 1 month [*β* = 1.70; 95% confidence interval (CI) 0.65; 2.76], regardless of the potential confounders included in the model. Stratification by parents' sex showed that this association was only significant for mothers (*β* = 1.99; 95% CI 0.70; 3.27) (Figure 2).

**TABLE 3:** Association between breastfeeding practices in the first and second generations of the 1993 Pelotas (Brazil) birth cohort study

Parent's predominant breastfeeding duration (months)	Exclusive breastfeeding duration (months)	
	Crude *β* (95% CI)^a^	Adjusted β (95% CI)^a^
Mothers and fathers^b^	*0.647*	*0.130*
<1	Ref.	Ref.
1–2.9	−0.08 (−0.58; 0.42)	0.15 (−0.38; 0.68)
≥3	0.24 (−0.48; 0.96)	0.67 (−0.12; 1.46)
Mothers^c^	*0.627*	*0.294*
<1	Ref.	Ref.
1–2.9	−0.14 (−0.70; 0.42)	−0.07 (−0.66; 0.52)
≥3	0.35 (−0.50; 1.20)	0.66 (−0.24; 1.57)
Fathers^c^	*0.866*	*0.263*
<1	Ref.	Ref.
1–2.9	0.11 (−0.94; 1.17)	1.20 (−0.06; 2.46)
≥3	0.11 (−1.25; 1.46)	0.93 (−0.70; 2.57)

Abbreviation: 95% CI, 95% confidence interval.

^a^ *P* values are shown for Tobit regression with exclusive breastfeeding duration censored at 6 months.

^b^ Adjusted for family income in 1993, grandmother's education and skin colour, maternal/paternal birthweight, age and sex, and second generation delivery type, preterm birth and birth weight.

^c^ Adjusted for family income in 1993, grandmother's education and skin colour, maternal/paternal birthweight and age, and second generation delivery type, preterm birth and birth weight.

**FIGURE 3:**
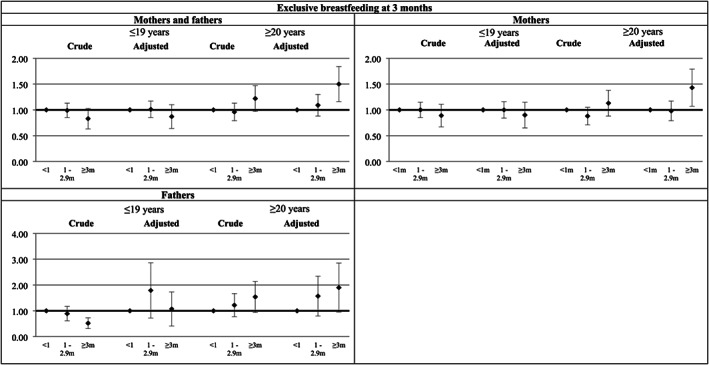
Association between first generation's predominant breastfeeding duration and second generation's exclusive breastfeeding at 3 months measured by Poisson regression. Note: adjusted for family income in 1993, grandmother's education and skin colour, maternal/paternal birthweight, age and sex, and second generation delivery type, prematurity, and birthweight

There was no association between parental predominant breastfeeding duration and prevalence of the second generation being exclusively breastfed at 3 and 6 months either in the crude or the adjusted models (Table 4). Nevertheless, stratification by parental age showed that being born to parents aged 20 years old or older and who had been predominantly breastfed for at least 3 months increased the prevalence of being exclusively breastfed for at least 3 months (PR = 1.50; 95% CI 1.16; 1.93), regardless of the confounders included in the adjusted model. These results were only statistically significant for mothers (PR = 1.43; 95% CI 1.07; 1.90) (Figure 3). On the other hand, parental predominant breastfeeding duration did not present any association with the prevalence of children still being exclusively breastfed at 6 months of age, even after stratification (results not shown in figures).

**TABLE 4:** Association between breastfeeding practices in the first and second generations of the 1993 Pelotas (Brazil) birth cohort study

Parentage breastfeeding duration (months)	Exclusive breastfeeding at 3 months		Exclusive breastfeeding at 6 months		
	Crude PR (95% CI)^a^	Crude PR (95% CI)^a^	Adjusted PR (95% CI)^a^	Adjusted PR (95% CI)^a^
Mothers and fathers^b^	*0.951*	*0.989*	*0.520*	*0.188*
<1	Ref.	Ref.	Ref.	Ref.
1–2.9	0.97 (0.86; 1.09)	0.92 (0.70; 1.21)	0.98 (0.73; 1.32)	1.03 (0.90; 1.18)
≥3	1.02 (0.86; 1.21)	1.04 (0.70; 1.52)	1.20 (0.78; 1.82)	1.16 (0.95; 1.40)
Mothers^c^	*0.815*	*0.871*	*0.813*	*0.543*
<1	Ref.	Ref.	Ref.	Ref.
1–2.9	0.94 (0.83; 1.07)	0.90 (0.67; 1.22)	0.88 (0.64; 1.21)	0.97 (0.84; 1.11)
≥3	1.00 (0.83; 1.22)	1.11 (0.73; 1.69)	1.14 (0.73; 1.79)	1.11 (0.90; 1.37)
Fathers^c^	*0.540*	*0.792*	*0.284*	*0.112*
<1	Ref.	Ref.	Ref.	Ref.
1–2.9	1.06 (0.76; 1.47)	0.97 (0.49; 1.92)	2.21 (0.79; 6.18)	1.50 (0.91; 2.48)
≥3	1.13 (0.76; 1.68)	0.88 (0.35; 2.19)	1.94 (0.52; 7.17)	1.55 (0.88; 2.72)

Abbreviations: 95% CI, 95% confidence interval; PR, prevalence ratio.

^a^ *P* values refer to Poisson regression with robust variance.

^b^ Adjusted for family income in 1993, grandmother's education and skin colour, maternal/paternal birthweight, age and sex, and second generation delivery type, preterm birth and birth weight.

^c^ Adjusted for family income in 1993, grandmother's education and skin colour, maternal/paternal birthweight and age, and second generation delivery type, preterm birth and birth weight.

Results comparing imputed and nonimputed data did not suggest any bias deriving from the imputation process. The only difference was observed in the Poisson regression stratified by parental age, when the prevalence of being exclusively breastfed at 3 months was the outcome: the results were only significant in the nonimputed scenario; the positive effect disappeared among those parents with imputed information for predominant breastfeeding duration (Tables S1 to S5).

## 4 DISCUSSION

This study aimed to evaluate intergenerational breastfeeding practices in the 1993 birth cohort study and showed an improvement in breastfeeding practices in the second generation. Exclusive breastfeeding duration in the second generation was almost 2 months longer than the predominant breastfeeding duration in the first generation (3.4 vs. 1.7 months). In addition, the prevalence of breastfeeding at 3 months of age between generations increased substantially, from 10% to more than 60%. These results are in consonance with the scientific literature, as an improvement in breastfeeding practices has been observed all over the world. In the Pelotas cohorts, for example, the prevalence of exclusive breastfeeding at 3 months of age increased from 7% in 1982 to 45% in 2015 (Santos et al., 2019).

In the current study, 20‐year‐old or older mothers who were predominantly breastfed for at least 3 months were 40% more likely to exclusively breastfed their children for at least 3 months. Other studies have also shown the role of maternal age on breastfeeding practices, indicating that older mothers tend to breastfeed their children for a longer period (Hauck, Fenwick, Dhaliwal, & Butt, 2011; Ogbo et al., 2017; Tang, Lee, & Binns, 2015). In contrast, a recent study conducted by Colombo et al. (2018) showed that 30‐year‐old or older mothers had higher odds of breastfeeding cessation when compared with younger mothers. However, none of these studies focused on intergenerational breastfeeding practices (Colombo et al., 2018).

Some studies report continuity of breastfeeding across generations (Meyerink & Marquis, 2002; Riva et al., 1999), and some mothers report that having a maternal grandmother who breastfed is influential in the decision to breastfeed (Ward, Sheridan, Howell, Hegarty, & O'Farrell, 2004). Notwithstanding, intergenerational continuity of breastfeeding has rarely been the primary focus of past research (Di Manno et al., 2015).

According to a systematic review, breastfeeding intention, initiation, duration and exclusivity are consistently associated with having been breastfed (Di Manno et al., 2015). We evaluated the duration of exclusive breastfeeding, and our findings are consistent only for those mothers over the age of 20, demonstrating that intergenerational patterns of breastfeeding may be age sensitive. This finding is consistent with a prospective study that reported a positive correlation between maternal age and breastfeeding duration. Additionally, the authors found that younger maternal age (≤19 years old) was associated with low birth weight [odds ratio (OR) 1.18 (95% CI 1.02; 1.36)], preterm birth [1.26 (1.03; 1.53)], stunting at 2 years [1.46 (1.25; 1.70)] and failure to complete secondary schooling [1.38 (1.18; 1.62)], compared with mothers aged 20–24 years. The authors referred that younger mothers have less maternal experience and autonomy. These features may lead to suboptimal nutrition, less adequate hygiene and healthcare seeking behaviours (Fall et al., 2015).

Different approaches used to measure breastfeeding duration limited our ability to make comparisons. In some studies that evaluated breastfeeding duration, the predictor variable was dichotomized, indicating whether participants had or had not been breastfed as infants (Brown, Raynor, & Lee, 2011; Forster, McLachlan, & Lumley, 2006; Meyerink & Marquis, 2002). Few studies measured breastfeeding duration in both generations (Berra et al., 2003; Horta et al., 2007). Berra et al. (2003) found that having been breastfed for less than 6 months was associated with a 27% risk of weaning before 6 months post‐partum. Horta et al. (2007) conducted the only Brazilian study on the topic and observed that infants whose adolescent mothers were breastfed for less than 1 month had an increased prevalence of 29% for stopping breastfeeding in the first 6 months of life. Moreover, the proportion of children who were totally breastfed for 6 months or more tended to increase as maternal duration of breastfeeding in infancy increased.

Previous studies regarding duration and exclusive breastfeeding evaluated these characteristics only among mothers. Even those studies that assessed intergenerational practices have focused solely on mother–child dyads. Our study differs from what is seen in other investigations as we have also added fathers to the analyses, despite the results not having been significant for them. According to the literature, males who were breastfed more often reported that they intended to support and/or encourage a partner to breastfeed (Di Manno et al., 2015). Additionally, it has also been demonstrated that women who initiated breastfeeding were more likely to report having a partner who was supportive of breastfeeding, irrespective of their own infant‐feeding status (Brown et al., 2011). This finding is important when considering social support surrounding breastfeeding, demonstrating that mothers can receive support for breastfeeding from their male partners, generating a positive environment conducive to breastfeeding (Brown et al., 2011; Vaaler et al., 2011).

Our study has strengths and weaknesses to be discussed. Different from previously published papers, our study also included fathers in the analyses, which allowed us to assess intergenerational breastfeeding practices in both sexes. In addition, we assessed the intergenerational effect of exclusive breastfeeding using a prospective design. Data on breastfeeding were prospectively collected in the first generation, which increases robustness for assessing causality. On the other hand, breastfeeding data for the second generation were cross‐sectionally gathered, which can be a limitation due to the recall bias involved in the gap between the children's age in the 22‐year‐old follow‐up and the age children stopped being exclusively breastfed. In our sample, the average gap was 34 months, ranging from 0.8 to 118 months.

Another limitation of our study is that information on predominant breastfeeding is only available for a subsample of parents, in which low birth weight parents are overrepresented. A multiple imputation procedure was carried out for 69% of the sample, and multiple imputation process shortcomings in such a large missing information set may cause the estimates to be biased. Final results were similar for imputed and nonimputed regression models, and this reduces the risk of bias in the imputed models.

In summary, an improvement was found in exclusive breastfeeding duration that may be partially attributed to an intergenerational breastfeeding practice, based on the finding that older mothers (**≥**20 years old) who were predominantly breastfed longer, tend to exclusively breastfeed their children for longer periods. This finding means that the positive effects of breastfeeding also appear to extend across generations. The study suggests that education and social support surrounding breastfeeding should be intensified among fathers, younger parents and health professionals to create a positive environment supportive of breastfeeding. It is important to strengthen the participation of health professionals and services, as they are essential to develop and actively work with breastfeeding support groups that comprised future mothers, fathers and grandparents.

## CONFLICTS OF INTEREST

The authors declare that they have no conflicts of interest.

## CONTRIBUTIONS

JSV and MCFA formulated the research question. ABM, MCFA and HG conceived and designed the study. LPS and APG analysed the data. JSV, LPS, GGD and IOB wrote the article. GK provided critical review.

## Supporting information


**Table S1.** Predominant breastfeeding duration of the first generation of the 1993 Pelotas (Brazil) birth cohort study according to imputation process.
**Table S2**. Adjusted association between breastfeeding practices in the first and second generations of the 1993 Pelotas (Brazil) birth cohort study, according to the imputation process.
**Table S3**. Adjusted association between breastfeeding practices in the first and second generations of the 1993 Pelotas (Brazil) birth cohort study, according to the imputation process.
**Table S4**. Association between breastfeeding practices in the first and second generations of the 1993 Pelotas (Brazil) birth cohort study, according to the imputation process.
**Table S5**. Association between breastfeeding practices in the 1^st^ and 2^nd^ generations of the 1993 Pelotas (Brazil) birth cohort study, according to the imputation process.

**Figure S1.** Acyclic Directed Graph (DAG) representing the hypothesis of relations between intergenerational breastfeeding practices. Among parents and their children.
Note: Red circles represent confounders variables; blue circles represent ancestors of the outcome; green circles represent ancestors of the exposure variable; dark pink arrows mean no casual path; green arrows mean causal paths; (1) represents cohort member characteristics; (2) represents second generation characteristics.
