# Supplementary material for: Intergenerational breastfeeding practices among parents and children: 1993 Pelotas (Brazil) birth cohort
Source: Matern Child Nutr. 2020 Jul 6;17(1):e13058. doi: 10.1111/mcn.13058 (PMC7729557; doi:10.1111/mcn.13058)
Supplement: Supplementary file 1 — Table S1. Predominant breastfeeding duration of the first generation of the 1993 Pelotas (Brazil) birth cohort study according to imputation process. Table S2. Adjusted association between breastfeeding practices in the first and second generations of the 1993 Pelotas (Brazil) birth cohort study, according to the imputation process. Table S3. Adjusted association between breastfeeding practices in the first and second generations of the 1993 Pelotas (Brazil) birth cohort study, according to the imputation process. Table S4. Association between breastfeeding practices in the first and second generations of the 1993 Pelotas (Brazil) birth cohort study, according to the imputation process. Table S5. Association between breastfeeding practices in the 1st and 2nd generations of the 1993 Pelotas (Brazil) birth cohort study, according to the imputation process. [file MCN-17-e13058-s002.docx]

**Imputation process**

**Supporting information table 1**. Predominant breastfeeding duration of the first generation of the 1993 Pelotas (Brazil) birth cohort study according to imputation process.

| **Variables** | **Predominant breastfeeding duration in months** | |
| --- | --- | --- |
|  | **Non-imputed (n=372)**  **Mean (95%CI)** | **Imputed**  **(n=518)**  **Mean (95%CI)** |
| **Parents’ characteristics (n=947)** | |  |
| Parentage | *0.667* | *0.006* |
| Mother | 1.75 (1.59; 1.91) | 1.61 (1.55; 1.68) |
| Father | 1.68 (1.38; 1.98) | 1.85 (1.67; 2.02) |
| Family income in 1993 (tertiles) | *0.029* | *0.005* |
| 1^st^ (lowest) | 1.80 (1.56; 2.03) | 1.55 (1.47; 1.65) |
| 2^nd^ | 1.43 (1.19; 1.68) | 1.62 (1.50; 1.73) |
| 3^rd^ (highest) | 1.90 (1.65; 2.14) | 1.79 (1.67; 1.92) |
| Age at delivery (years) | *0.736* | *0.562* |
| ≤ 19 | 1.76 (1.55; 1.90) | 1.67 (1.58; 1.76) |
| $\geq$20 | 1.71 (1.58; 1.76) | 1.64 (1.55; 1.72) |
| Low birth weight (<2,500 g) | *0.128* | *0.340* |
| No | 1.83 (1.65; 2.02) | 1.65 (1.59; 1.71) |
| Yes | 1.61 (1.37; 1.84) | 2.00 (0.48; 3.52) |
|  |  |  |
| **Total*** | **1.73 (1.59; 1.87)** | **1.65 (1.59; 1.72)** |

95% CI = 95% confidence interval; *P*-values refer to Analysis of Variance (ANOVA). **P*-value = 0.271 for the difference between non-imputed and imputed predominant breastfeeding duration

**Supporting information table 2**. Adjusted association between breastfeeding practices in the first and second generations of the 1993 Pelotas (Brazil) birth cohort study, according to the imputation process.

| **Parent’s predominant breastfeeding duration (months)** | **Exclusive breastfeeding duration (months)** | |
| --- | --- | --- |
|  | **Non-imputed (n=372)**  **Adjusted β (95% CI) ^†^** | **Imputed (n=518)**  **Adjusted β (95% CI) ^†^** |
| Mothers and fathers ^‡^ | *0.365* | *0.678* |
| <1 | ref. | ref. |
| 1 - 2.9 | -0.16 (-1.03; 0.71) | -0.12 (-0.82; -0.58) |
| ≥ 3 | 0.48 (-0.47; 1.43) | 0.77 (-0.64; 2.19) |
|  |  |  |
| Mothers ^3^ | *0.791* | *0.752* |
| <1 | ref. | ref. |
| 1 - 2.9 | -0.52 (-1.48; 0.44) | -0.25 (-1.01; 0.51) |
| ≥ 3 | 0.22 (-0.85; 1.30) | 1.23 (-0.44; 2.91) |
|  |  |  |
| Fathers ^§^ | *0.245* | *0.562* |
| <1 | ref. | ref. |
| 1 - 2.9 | 0.07 (-2.17; 2.30) | 0.96 (-0.90; 2.81) |
| ≥ 3 | 1.12 (-0.88; 3.13) | 0.69 (-2.11; 3.49) |
|  |  |  |

95% CI = 95% confidence interval; **^†^***P*-values are shown for Tobit regression with exclusive breastfeeding duration censored at 6 months; ^‡^Adjusted for family income in 1993, grandmother’s education and skin color, maternal/paternal birthweight, age and sex, and second generation delivery type, prematurity and birth weight. ^§^Adjusted for family income in 1993, grandmother’s education and skin color, maternal/paternal birthweight and age, and second generation delivery type, prematurity and birth weight.

**Supporting information table 3**. Adjusted association between breastfeeding practices in the first and second generations of the 1993 Pelotas (Brazil) birth cohort study, according to the imputation process.

| **Parent’s predominant breastfeeding duration (months)** | **Exclusive breastfeeding duration (months)** | | | |
| --- | --- | --- | --- | --- |
|  | **≤19 years** | | **≥20 years** | |
|  | **Non-imputed (n=372)**  **Adjusted β (95% CI) ^†^** | **Imputed (n=518)**  **Adjusted β (95% CI) ^†^** | **Non-imputed (n=372)**  **Adjusted β (95% CI) ^†^** | **Imputed (n=518)**  **Adjusted β (95% CI) ^†^** |
| Mothers and fathers ^‡^ | *0.540* | *0.418* | *0.037* | *0.100* |
| <1 | ref. | ref. | ref. | ref. |
| 1 - 2.9 | 0.25 (-0.99; 1.50) | -0.56 (-1.58; 0.46) | -0.26 (-1.40; 0.86) | 0.46 (-0.51; 1.43) |
| ≥ 3 | -0.54 (-1.95; 0.86) | -0.39 (-2.33; 1.54) | 1.35 (0.14; 2.56) | 2.18 (0.09; 4.28) |
|  |  |  |  |  |
| Mothers ^§^ | *0.379* | *0.574* | *0.061* | *0.247* |
| <1 | ref. | ref. | ref. | ref. |
| 1 - 2.9 | 0.17 (-1.16; 1.50) | -0.58 (-1.65; 0.50) | -0.79 (-2.08; 0.50) | 0.23 (-0.87; 1.33) |
| ≥ 3 | -0.80 (-2.32; 0.72) | 0.09 (-2.07; 2.25) | 1.51 (0.09; 2.93) | 3.04 (0.07; 6.02) |
|  |  |  |  |  |
| Fathers ^§^ | *0.168* | *0.430* | *0.299* | *0.240* |
| <1 | ref. | ref. | ref. | ref. |
| 1 - 2.9 | 0.04 (-2.26; 2.35) | 3.76 (-3.79; 11.30) | -0.71 (-3.49; 2.08) | 1.08 (-1.15; 3.30) |
| ≥ 3 | -1.51 (-3.82; 0.79) | 0.54 (-6.86; 7.94) | 1.03 (-1.37; 3.43) | 2.10 (-1.52; 5.73) |
|  |  |  |  |  |

95% CI = 95% confidence interval; **^†^** *P*-values are shown for Tobit regression with exclusive breastfeeding duration censored at 6 months; ^‡^Adjusted for family income in 1993, grandmother’s education and skin color, maternal/paternal birthweight, age and sex, and second generation delivery type, prematurity and birth weight. ^§^ Adjusted for family income in 1993, grandmother’s education and skin color, maternal/paternal birthweight and age, and second generation delivery type, prematurity and birth weight.

**Supporting information table 4**. Association between breastfeeding practices in the first and second generations of the 1993 Pelotas (Brazil) birth cohort study, according to the imputation process.

| **Parentage breastfeeding duration (months)** | **Exclusive breastfeeding at 3 months** | |
| --- | --- | --- |
|  | **Non-imputed (n=372)**  **Adjusted PR (95% CI) ^†^** | **Imputed (n=518)**  **Adjusted PR (95% CI) ^†^** |
| Mothers and fathers ^‡^ | *0.090* | *0.465* |
| <1 | ref. | ref. |
| 1 - 2.9 | 1.04 (0.82; 1.33) | 0.91 (0.78; 1.06) |
| ≥ 3 | 1.24 (0.97; 1.58) | 0.99 (0.71; 1.39) |
|  |  |  |
|  | **≤19 years** | |
| Mothers and fathers ^‡^ | *0.927* | *0.078* |
| <1 | ref. | ref. |
| 1 - 2.9 | 1.15 (0.85; 1.54) | 0.83 (0.67; 1.03) |
| ≥ 3 | 0.98 (0.69; 1.40) | 0.71 (0.43;1.16) |
|  |  |  |
|  | **≥20 years** | |
| Mothers and fathers ^‡^ | *0.016* | *0.478* |
| <1 | ref. | ref. |
| 1 - 2.9 | 0.97 (0.65; 1.46) | 1.00 (0.80; 1.26) |
| ≥ 3 | 1.55 (1.08; 2.21) | 1.41 (0.90; 2.21) |

PR = Prevalence ratio; 95% CI = 95% confidence interval; **^†^** *P*-values refer to Poisson regression with robust variance; ^‡^Adjusted for family income in 1993, grandmother’s education and skin color, maternal/paternal birthweight, age and sex, and second generation delivery type, prematurity and birth weight.

**Supporting information table 5**. Association between breastfeeding practices in the 1^st^ and 2^nd^ generations of the 1993 Pelotas (Brazil) birth cohort study, according to the imputation process.

| **Parentage breastfeeding duration (months)** | **Exclusive breastfeeding at 6 months** | |
| --- | --- | --- |
|  | **Non-imputed (n=372)**  **Adjusted PR (95% CI) ^†^** | **Imputed (n=518)**  **Adjusted PR (95% CI) ^†^** |
| Mothers and fathers ^‡^ | *0.717* | *0.288* |
| <1 | ref. | ref. |
| 1 - 2.9 | 0.77 (0.45; 1.26) | 1.02 (0.69; 1.49) |
| ≥ 3 | 0.93 (0.56; 1.55) | 1.89 (0.99; 3.58) |
|  |  |  |
|  | **≤19 years** | |
| Mothers and fathers ^‡^ | *0.429* | *0.765* |
| <1 | ref. | ref. |
| 1 - 2.9 | 0.98 (0.52; 1.84) | 0.77 (0.46; 1.29) |
| ≥ 3 | 0.74 (0.36; 1.51) | 1.20 (0.47; 3.02) |
|  |  |  |
|  | **≥20 years** | |
| Mothers and fathers ^‡^ | *0.741* | *0.039* |
| <1 | ref. | ref. |
| 1 - 2.9 | 0.58 (0.27; 1.24) | 1.51 (0.83; 2.77) |
| ≥ 3 | 1.21 (0.52; 2.77) | 3.41 (1.39; 8.37) |

PR = Prevalence ratio; 95% CI = 95% confidence interval; **^†^** *P*-values refer to Poisson regression with robust variance; ^‡^Adjusted for family income in 1993, grandmother’s education and skin color, maternal/paternal birthweight, age and sex, and second generation delivery type, prematurity and birth weight.
