# Supplementary material for: Intergenerational breastfeeding practices among parents and children: 1993 Pelotas (Brazil) birth cohort
Source: Matern Child Nutr. 2020 Jul 6;17(1):e13058. doi: 10.1111/mcn.13058 (PMC7729557; doi:10.1111/mcn.13058)
Supplement: Supplementary file 2 — Figure S1. Acyclic Directed Graph (DAG) representing the hypothesis of relations between intergenerational breastfeeding practices. Among parents and their children. Note: Red circles represent confounders variables; blue circles represent ancestors of the outcome; green circles represent ancestors of the exposure variable; dark pink arrows mean no casual path; green arrows mean causal paths; (1) represents cohort member characteristics; (2) represents second generation characteristics. [file MCN-17-e13058-s001.docx]

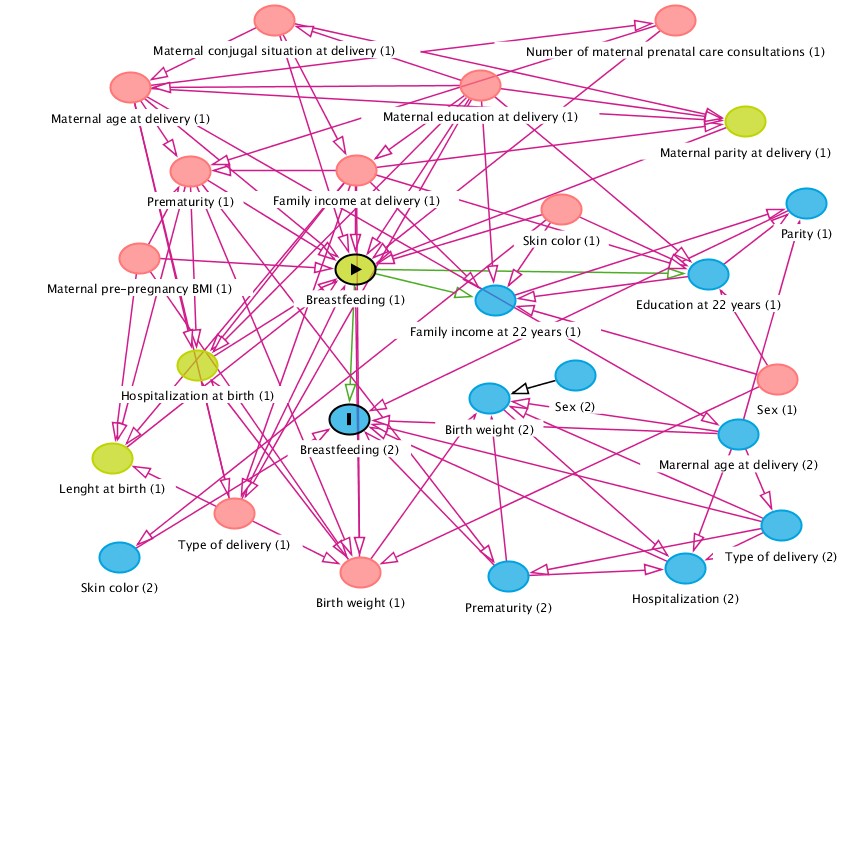


**Supporting Information Figure 1.** Acyclic Directed Graph (DAG) representing the hypothesis of relations between intergenerational breastfeeding practices. among parents and their children.

Note: Red circles represent confounders variables; blue circles represent ancestors of the outcome; green circles represent ancestors of the exposure variable; dark pink arrows mean no casual path; green arrows mean causal paths; (1) represents cohort member characteristics; (2) represents second generation characteristics.
